# A review of the MRI features of endometriosis: what should be paid attention to during the reporting process?

**DOI:** 10.1007/s00261-025-05002-x

**Published:** 2025-05-29

**Authors:** Yesim Yekta Yuruk, Merve Sam Ozdemir, Mehmet Simsar, Hilal Sahin

**Affiliations:** 1 University of Health Sciences, Izmir Tepecik Training and Research Hospital, Izmir, Turkey; 2 Izmir City Hospital, Izmir, Turkey; 3 Basaksehir Cam and Sakura City Hospital, Istanbul, Turkey; 4 Izmir City Hospital, Izmir, Turkey; 5 University of Health Sciences, Izmir Faculty of Medicine, Izmir, Turkey

**Keywords:** Endometriosis, Magnetic resonance imaging, Diagnosis, Education

## Abstract

Endometriosis is a chronic gynecological disorder characterized by the ectopic presence of endometrial tissue, often resulting in pelvic pain, infertility, and decreased quality of life. Magnetic Resonance Imaging (MRI) plays a crucial role in noninvasive diagnosis and preoperative assessment of endometriosis, particularly in evaluating complex or deep infiltrative diseases. A detailed and structured report on lesion depth, extension, and involvement of critical anatomical structures is vital for multidisciplinary teams’ decision-making. By comprehensively understanding and recognizing the complete range of endometriosis manifestations, radiologists can significantly enhance individualized treatment strategies and improve patient outcomes. This pictorial review highlights the key MRI features of endometriosis and provides essential guidance for radiologists during the imaging and reporting process.

## Introduction

Endometriosis, which is a chronic inflammatory gynecological disorder characterized by the presence of active endometrial glands and mucosa outside the uterine cavity, affects approximately 10% [1] of women in reproductive age and is found in 20–50% of women with infertility [2–4] and affects about 90% of women with chronic pelvic pain [1–5].

The primary mechanism that causes disease is the presence of endometrial tissue outside the uterine cavity, where cyclical proliferative activity occurs without menstrual bleeding, followed by lysis, bleeding, and regeneration linked to proliferation and/or fibrosis [6].

There are various theories regarding its pathophysiology. One of these is Meyer’s coelomic metaplasia theory, which is based on the potential for differentiation of mesothelium. Consequently, undifferentiated cells located within the peritoneal layers may undergo transformation into ectopic endometrial cells as a direct result of endometrial induction [7]. On the other hand, Sampson’s metastatic theory explains the pathophysiology of endometriosis, as possible reflux of living endometrial cells during menstruation, along with implantation, hematogenous, and lymphatic spread [8].

Endometriosis is classified into two categories as pelvic and extrapelvic. Furthermore, three primary entities of pelvic endometriosis have been identified. In superficial endometriosis, endometrial tissue implants invade peritoneum to a depth of less than 5 mm, making visualization of the lesion difficult. In endometriomas, thick-walled cystic ovarian lesions, generally known as chocolate cysts, contain hemorrhagic and dense proteinaceous products. In deep infiltrating endometriosis, endometrial tissue implants invade peritoneum more than 5 mm [9].

Accurate identification of lesions and evaluation of the adhesions’ severity are vital in guiding treatment decisions. Imaging modalities significantly have a crucial role in this process. Ultrasound is frequently used as the first-line imaging modality when endometriosis is suspected. However, MRI provides a more accurate assessment of complex diseases. Pre-operative MRI is especially beneficial for diagnosing endometriosis and characterizing extent of disease, playing a key role in guiding surgical management [5, 10].

### Optimal MRI protocols for endometriosis

The initial step in MRI reporting of endometriosis involves utilization of appropriate MRI protocols. MRI is performed with a 1.5 T or 3 T scanner and high-resolution phased array coils. However, low magnetic fields or open MRIs are not recommended because they lack adequate image quality. Imaging of the pelvis is unaffected by the phase of menstrual cycle [11, 12].

T1 weighted fat-saturated (T1 W-FS) axial and sagittal images are used to evaluate for endometrial foci and superficial endometriosis. T2 weighted (T2 W) axial, sagittal, and coronal images are used to evaluate for deep infiltrating endometriosis. Both T2 W and T1 W-FS images are used to evaluate for endometriomas. If a malignancy or infection developing due to endometriosis is suspected, a contrast-enhanced T1 W or diffusion weighted image (DWI)-apparent diffusion coefficient (ADC) images can be used. However, DWI-ADC or contrast-enhanced images alone do not have the ability to diagnose endometriosis [11, 13]. There are also various suggestions to prevent artifacts and improve image quality. Anti-peristaltic agents are highly recommended to minimize bowel motion artifacts, moderate bladder distension, and 3–6 h fasting before imaging is recommended [11, 13].

There is no consensus in literature regarding the usefulness of vaginal and rectal opacification. However, there are various publications that find them useful for detecting DIE [11, 14, 15].

## Pelvic endometriosis

### Superficial peritoneal endometriosis

Superficial peritoneal endometriosis (SPE) is described as implants less than 5 mm in depth that cause reactive proliferation of stromal vessels, leading to recurrent bleeding. Recurrent bleeding and inflammation lead to fibrosis and hemosiderin deposition, which is characterized as a powder burn lesion [16]. These SPE implants may appear as hyperintense foci on T1 WI. T1 W-FS axial and sagittal images are described as the primary sequences for evaluating SPE [13]. Additionally, these lesions may present as millimetric peritoneal pseudocysts [17] or adhesions [18]. Adhesions extending between pelvic organs may appear as low signal intensity bands of varying thickness on T2-weighted images in cases of long-standing endometriosis [18].

SPE implants may be complex to identify on MRI unless they are hemorrhagic and hyperintense on T1 WI. Using cinematic images in MRI protocols may identify indirect findings of SPE implants, such as decreased or fixed bowel motility, and deterioration detected on still images with 97% sensitivity (95%, confidence interval 82–100%) [19, 20].

### Endometrioma

Endometriomas, also known as chocolate cysts, are thick-walled cysts that contain degenerated blood products. They may develop in various locations within the pelvis, most frequently in ovaries (50% bilateral) [9].

MRI plays a significant role in detecting endometriomas, especially when they appear distinctive imaging features. The principal imaging findings on MRI for diagnostic purposes include presence of T1 high-signal multiplicity, T2 shading (93% sensitivity), and T2 dark spot sign (93% specificity) [21–23].

T1 high-signal multiplicity occurs as a result of repeated bleeding and formation of new blood locules, and it appears as multiple hyperintense cysts on T1 WI [21].

**Fig. 1:**
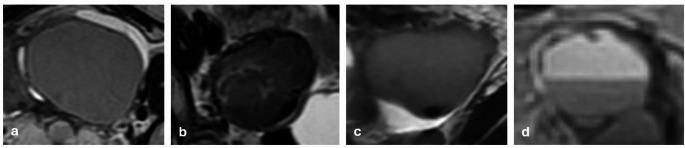
T2 shading patterns in endometriomas. Homogeneous (**a**), heterogeneous (**b**), layering (**c**), fluid-fluid level (**d**)

T2-shading sign is described as decreased signal intensity (SI) on T2 WI. It shows chronic repeated bleeding with high concentrations of iron and protein within the cyst. This sign has different patterns, such as homogeneous (the most common), heterogeneous (the most specific), layering, and fluid-fluid level (the least common) [21, 22] (Fig. 1).

**Fig. 2:**
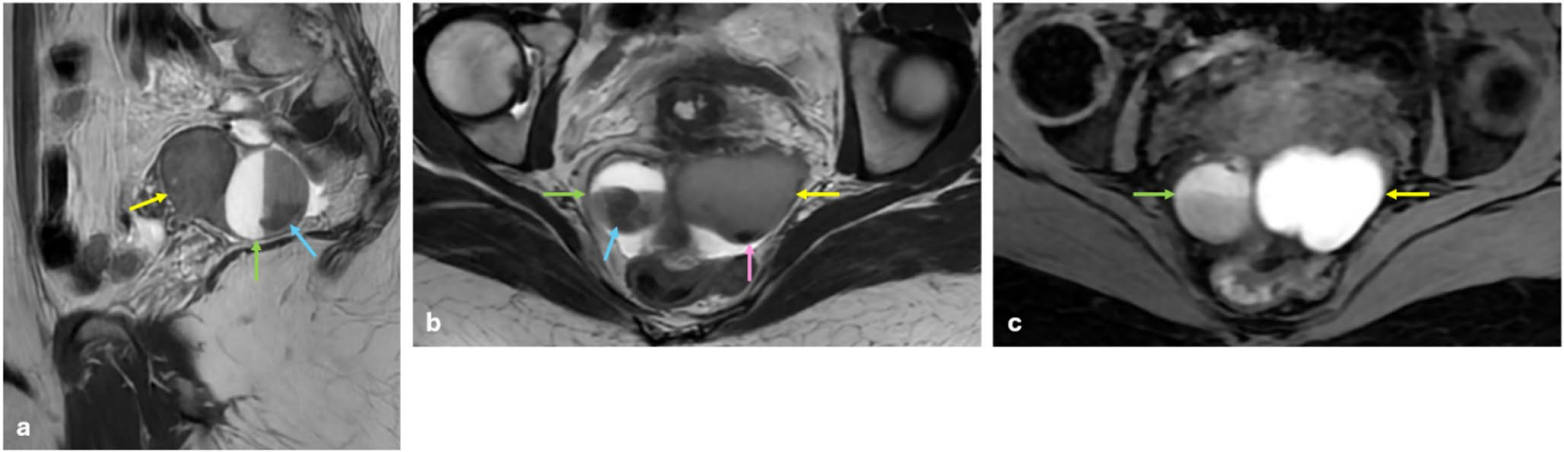
Endometrioma and hemorrhagic cyst. In the right ovary, a 3 cm hemorrhagic cyst is shown that has fluid-fluid leveling on sagittal (**a**) and axial (**b**) T2 W images (green arrows), containing clot product (blue arrows) and hyperintense signal on fat-saturated T1 WI (**c**). In the left ovary, a 4.5 cm endometrioma is shown that has hypointense signal on sagittal (**a**) and axial (**b**) T2 W images (yellow arrows) with T2 dark spot sign (pink arrow) and bright hyperintense signal on fat-saturated T1 WI (**c**). Clot products, which are found in hemorrhagic cysts, may be confused with a T2 dark spot sign. The major difference from the T2 dark spot sign is that clot products have sharp or concave borders. Hemorrhagic cysts may have variable signals on T1 WI, but endometriomas always appear bright hyperintense on T1 WI

T2 dark-spot sign is the most specific sign for detecting ovarian endometriomas, particularly for differentiating them from functional ovarian hemorrhages. It appears as well-defined, hypointense foci within the cyst due to chronic retracted blood products with high concentrations of protein and hemosiderin [22, 23] (Fig. 2).

**Fig. 3:**
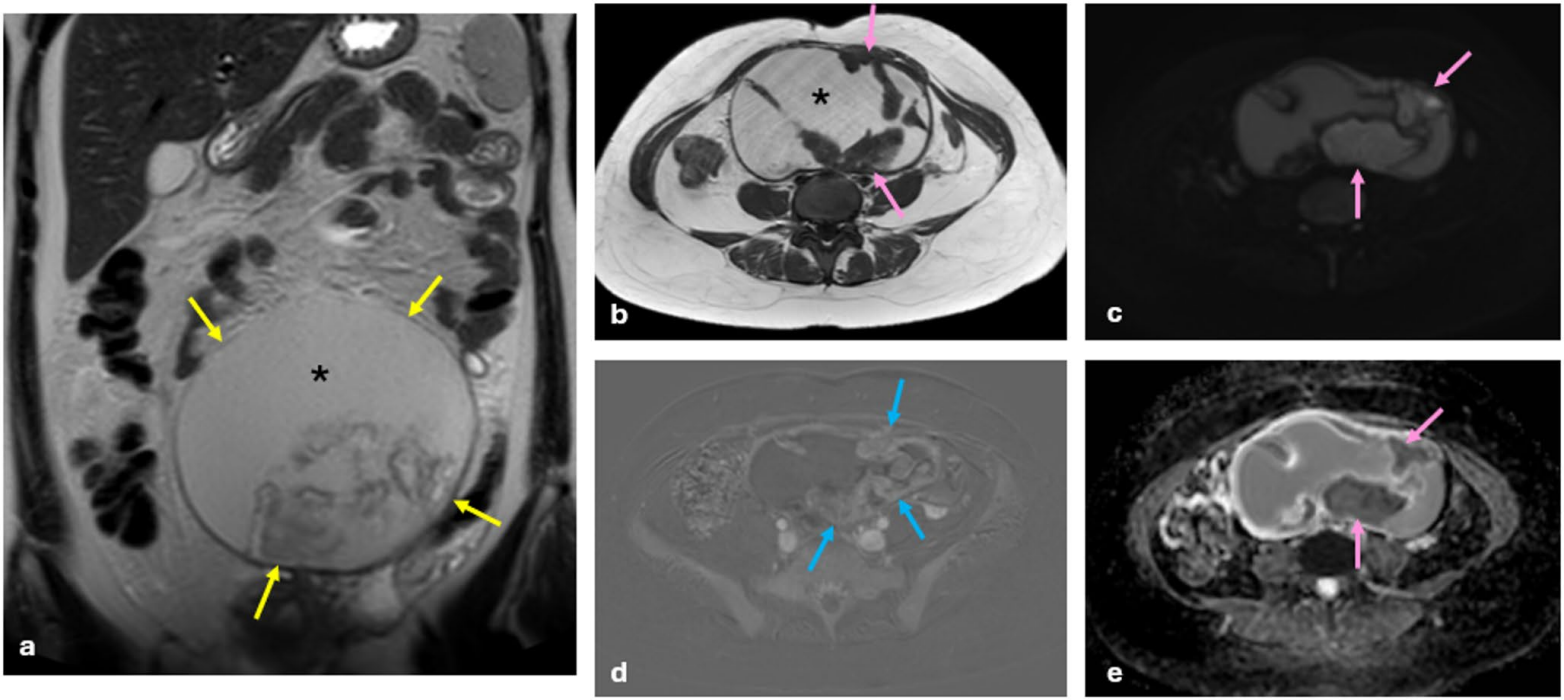
Endometrioma with suspicious features for malignancy. A patient, who was known to be followed up with a history of left endometrioma for many years, underwent an MRI after a giant cystic mass containing papillomatous protrusions was observed on the last ultrasonography. On MRI, a giant cystic mass (yellow arrows), approximately 20 cm in size extending from the pelvic midline to the upper abdomen with papillomatous protrusions on its inside wall is observed on the coronal T2 WI (**a**). The fluid inside this cystic mass (asterisks) is hyperintense on the T1 WI (**b**) and shows intermediate signal on the T2 WI (**a**). Papillomatous protrusions on the cyst wall (**b**) restrict diffusion (**c**) and show low ADC characteristics (**e**) (pink arrows). The subtraction image (**d**) of contrast-enhanced fat-saturated T1 WIs clearly shows that papillary projections are contrast-enhanced (blue arrows). All these findings, with the patient’s history, are suspicious for malignancy and suggest that it may develop based on endometriosis. The outcome of the specimen obtained from the mass has been identified as an endometrioid ovarian borderline tumor, thereby confirming our preliminary diagnosis

Although the likelihood is low, endometriotic cysts are associated with malignancy and described as endometriosis-associated ovarian carcinoma (EAOC). The most common histological types of EAOC are endometrioid adenocarcinoma and clear cell carcinoma. Infrequently, benign and borderline seromucinous tumors have also been observed [9]. The most significant indicator of malignancy is the appearance of enhanced mural nodules within the ovarian endometriotic cyst (present in 97% of EAOCs) [13, 24]. Another important finding for malignancy is the increase in size of the lesion. In the study conducted by Nishio et al., it was observed that lesions developing malignancy increased almost twofold in size, while there was no such increase in size in the control group [25]. The lack of a T2-shading sign may also be a notable finding for malignancy [24]. T2-shading was present in 81.3% of patients with benign conditions, compared to 33.3% in EAOC [24–26] (Fig. 3).

### Deep infiltrative endometriosis

Deep infiltrative endometriosis (DIE) is described as implants that invade the peritoneum more than 5 mm. As a result of this invasion, solid lesions occur as a combination of glandular, stromal, and/or fibrotic tissue. On MRI, imaging features of lesions are based on the lesions’ characteristics. Active glandular tissues contain hemorrhagic and/or proteinaceous formations. These formations cause lesions that are hyperintense on T1 WI and hypointense on T2 WI. On the other hand, chronic stromal-fibrotic tissues, as the name suggests, contain mainly fibrosis, and also hypertrophy of smooth muscle. As a result of fibrosis, these lesions are seen as linear and/or stellate and may extend into periregional structures, such as the uterus, ovaries, or rectum. On MRI, these fibrotic tissues are seen as hypointense to muscle on T2 WI. Moreover, the lesions that extend into periregional structures may be seen as hypointense on either T2 W or T1 W images [27].

**Fig. 4:**
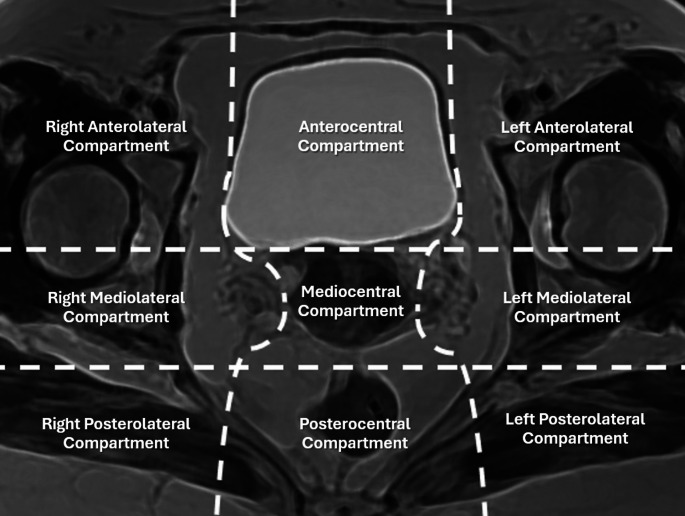
Illustration of the nine pelvic compartments proposed by the ENDOVALIRM group. In this illustration, inspired by the T2-weighted MR image in the axial plane at the USL level, the pelvis is divided into nine parts according to the suggested anatomical structures in lexicon. Two horizontal lines (horizontal dashed lines) divide the pelvis into anterior, median, and posterior parts. Anterior horizontal line passes through the anterior to the cervix or vagina. Posterior horizontal line passes through the anterior to the rectum. Two vertical lines (vertical dashed lines) divide the pelvis into a central and two lateral parts. These vertical lines extend from back to front through the USLs and the underlying fascia recti, lateral borders of uterine cervix or vagina, and lateral walls of the bladder

There are certain guidelines and lexicons that have been presented for imaging DIE. The rASRM (revised American Society for Reproductive Medicine) classification, one of the most well-known and widely used scoring systems worldwide, aims to score endometriosis foci according to their depth in the ovary and peritoneum, the formation of adhesions in the ovary and tubes, and posterior cul-de-sac obliteration [28]. The disadvantage of the rASRM classification is that it does not include the presence of DIE in different sites, such as the uterosacral ligaments, bladder, or bowel. Therefore, the ENZIAN classification was developed to evaluate DIE in retroperitoneal structures. In the ENZIAN classification, DIE scoring is calculated using three compartments: A, vagina-rectovaginal space (RVS); B, uterosacral ligaments (USL), cardinal ligaments, pelvic sidewall; C, rectum. Apart from these three compartments, it also includes other compartments: F, far locations such as urinary bladder (FB), the ureters (FU), other intestinal locations (FI), and other extragenital lesions (FO); P, peritoneal involvement; O, ovary; T, tubo-ovarian lesions. Individual compartments or organ involvement are identified with capital letters and arranged in this order. Paired organs are arranged separately after the letter. Missing/invisible ovary or tube is described with suffixes such as m, missing; x, unknown. However, the ENZIAN classification does not include peritoneal or ovarian disease or adhesions [29]. In 2020, the Society of Abdominal Radiology (SAR) Endometriosis Disease-Focused Panel published an MRI lexicon for endometriosis MRI evaluation and anatomic localization. In this lexicon, the pelvis is divided into three compartments: anterior, middle, and posterior. Although DIE lesions are well described, and lesions outside the pelvis have also been defined within the extrapelvic compartments, lateral compartment structures have not been specified separately [19]. The ENDOVALIRM group published the MRI consensus lexicon and compartment-based approach in 2023. In this approach, the pelvis is divided into nine compartments and structures within each compartment are described in detail [30] (Fig. 4).

### Anterolateral compartment

Anterolateral compartment contains distal round ligament (distal two-thirds of intrapelvic part) [30]. Ligaments affected by DIE show asymmetric features such as thickening, shortening, and nodularity with hypointense signal intensity on both T1 W and T2 W images due to fibrosis [27, 30].

### Anterocentral compartment

Anterocentral compartment contains proximal round ligaments (proximal one-thirds of intrapelvic part) and bladder [30].

**Fig. 5:**
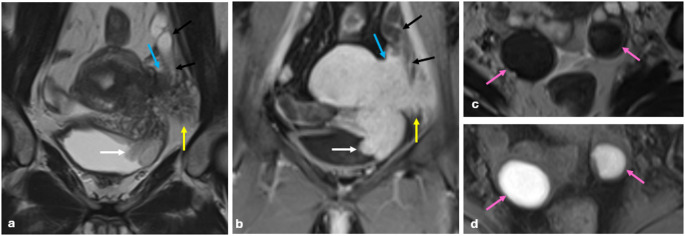
Deep infiltrating endometriosis with lateral pelvic compartment involvement and bilateral ovarian endometriomas. On MRI, an infiltrative lesion is shown that is extended from the left anterior part of the uterus to the proximal left ureter (blue arrows) and causing ureteral dilatation (black arrows), infiltrating the bladder wall and causing a polypoid lesion in the bladder lumen (white arrows), extending to the lateral compartment (yellow arrows), infiltrating the surrounding tissue with hypointense fibrous bands on T2 WI (**a**), and is observed as hyperintense on contrast-enhanced fat-saturated T1 WI (**b**) is detected. Additionally, bilateral ovarian endometriomas that are observed as hypointense on T2 WI (**c**) and hyperintense on fat-saturated T1 WI (**d**) series are detected. All these findings suggest DIE involvement in the bladder, uterus, and ureter. The outcome of the specimen obtained from the polypoid lesion within the bladder during the transurethral resection procedure has been identified as external endometriosis, thereby confirming our diagnosis

Bladder involvement is a rare condition for DIE (< 1%) [31, 32]. The posterior wall of bladder is the most commonly affected area. Bladder endometriosis is caused by an infiltrating fibrous reaction that extends into the bladder muscularis layer, infrequently extending with full thickness to mucosal layer. As a result, localized thickening and irregular borders may occur on bladder wall. DIE lesions are frequently isointense to bladder wall on T2 W images and may contain T1 W hyperintense foci. In cases where detrusor muscle is infiltrated full thickness, it may manifest as a mural mass that protrudes into bladder lumen [32]. These patients may present with hematuria, which may mimic malignancy (Fig. 5).

### Mediolateral compartment

Mediolateral compartment contains parametrium, ureter, uterine artery, external iliac and obturator vessels [30].

Parametrium is generally described as a band-like connective tissue that contains blood vessels, ureter, and inferior hypogastric plexus. It extends from the lateral surface of the cervix and vagina to the lateral pelvic wall in the frontal plane. Parametrium involvement of DIE may present as irregular, retractive fibrotic infiltration and/or hemorrhagic cystic areas [30] (Fig. 5a and b).

Ureteral endometriosis occurs more often on the left side due to peritoneal flow blockage caused by the sigmoid colon. Among the ureteral parts, the distal ureter is located in the anterior compartment and is also the most commonly affected part of DIE. Moreover, ureteral dilatation, an indirect manifestation, may be the only sign of involvement in DIE. Endometriotic lesions may also originate from within the muscularis and lamina propria of ureter, or they may occur due to lesions developing in structures close to ureter and result from wrapping around ureter. On MRI, endometriosis lesions are seen as hypointense nodules with spiculated margins on T2 WI with/without hyperintense foci on T1 WI. In urinary tract involvement of DIE, accurately defining the depth of muscle invasion, location, and distance from ureteral meatus in MRI report is crucial for both treatment and surgical planning [30, 33].

The degree of DIE involvement in mediolateral compartment must be specified individually, as recording all affected structures, including the vascular and nerve structures, will change the surgical method and outcome [30, 34].

### Mediocentral compartment

The ENDOVALIRM group defines the mediocentral compartment as it includes the torus uterinum with proximal USL (< 2 cm from the torus uterinum), posterior vaginal fornix, rectovaginal septum (RVS) and anterior mesorectum, anterior and/or posterior external adenomyosis [30]. Positive diagnosis of DIE involvement in torus uterinum and proximal USL is described by ENDOVALIRM group as follows: regular or irregular fibrotic thickening (> 5 mm) and/or nodular and/or retractions or presence of hemorrhagic implants [30]. However, this mediocentral compartment definition lacks internal genital organs such as the uterus, vagina, and fallopian tubes, and these areas are also affected by DIE. On the contrary, in the SAR lexicon, these structures are defined in the middle compartment, and how they are affected by DIE is explained [19].

**Fig. 6:**
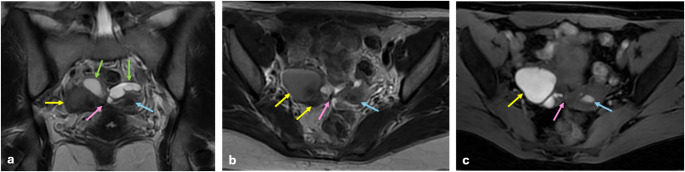
Deep infiltrating endometriosis with kissing ovaries sign. On coronal T2 W (**a**), axial T2 W (**b**), and axial fat-saturated T1 W (**c**) images show a 4 cm right ovarian endometrioma (yellow arrows) and a 1 cm left ovarian endometrioma (blue arrows) with hydrosalpinx (green arrows). Bilateral ovaries are medialized and touch each other (pink arrows) in the rectouterine pouch. This sign is known as ‘kissing ovaries’

Ovarian endometriomas significantly increase the risk associated with DIE, although they are not classified within DIE. Paraovarian endometriotic lesions commonly occur as fibrotic band-like lesions that extend into the uterus and/or other ovary. Consequently, the two ovaries approach one another, become medialized, and establish contact as a result of fibrous bands, leading to the phenomenon commonly referred to as “kissing ovaries” sign (Fig. 6). This sign strongly suggests moderate to severe endometriosis [13, 19].

**Fig. 7:**
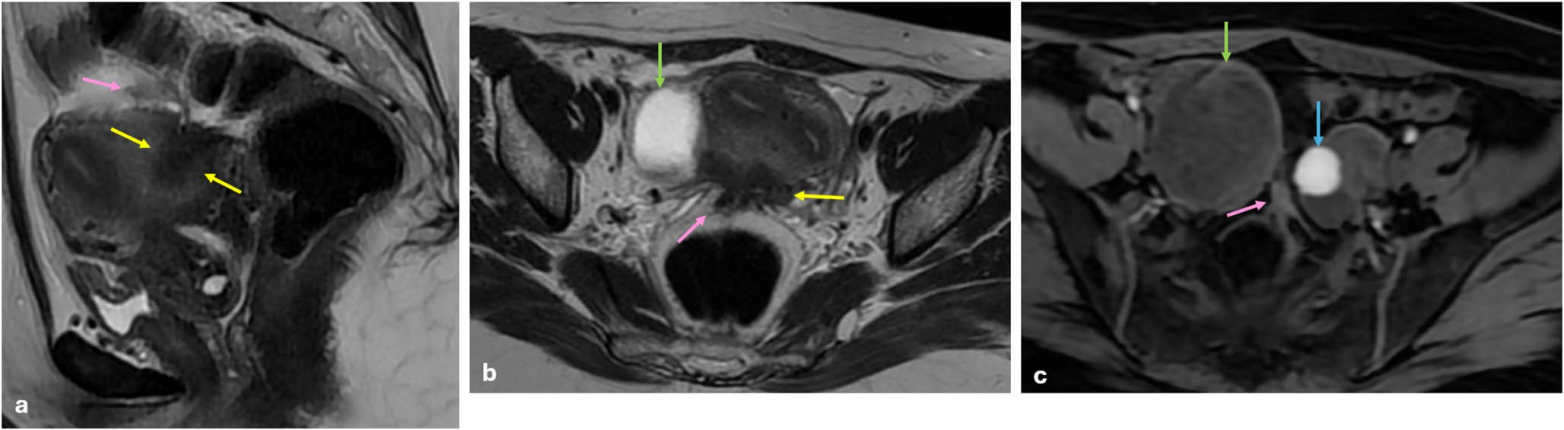
Torus uterinum involvement and ovarian endometrioma. On sagittal (**a**) and axial T2 W (**b**) images show a hypointense infiltrative lesion and an arciform abnormality on the torus uterinum (yellow arrows), which extends fibrous bands to peripheral fat tissue and through the rectosigmoid colon wall (pink arrows) on both sagittal-axial T2 W and fat-saturated T2 W (**c**) images. The torus uterinum involvement with distal uterosacral ligament involvement may result in an arciform abnormality on the uterus. Additionally, there is a 4 cm simple cyst (green arrows) in the right ovary and a 2 cm endometrioma (blue arrow)

**Fig. 8:**
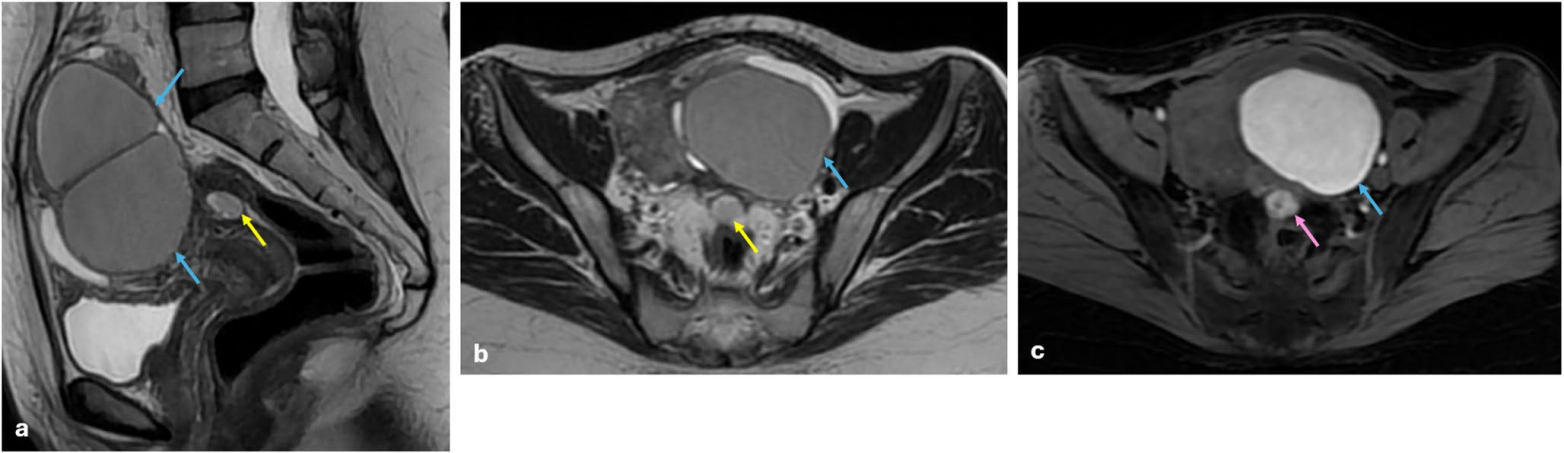
Torus uterinum involvement and ovarian endometrioma. On sagittal (**a**) and axial T2 W (**b**) images show a hypointense (yellow arrows), and axial fat-saturated T1 WI (**c**) shows a hyperintense (pink arrow) 2 cm cystic endometriotic lesion on the torus uterinum with no major infiltration to neighboring structures. Torus uterinum endometriosis may occur in cystic or infiltrative form. Additionally, there is a 7 cm endometrioma on the left ovary (blue arrows)

**Fig. 9:**
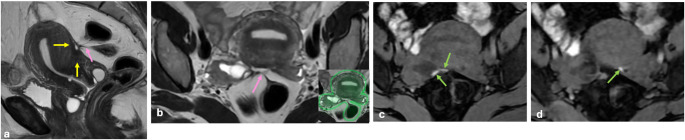
Deep infiltrating endometriosis with cloverleaf sign. On sagittal (**a**) and axial T2 W (**b**) images show a T2 hypointense infiltrative plaque in the torus uterinum (yellow arrows), extending into the rectum wall and bilateral ovaries as fibrous bands. The appearance of the uterus, ovaries, and rectum being pulled together by these fibrous bands is known as the ‘cloverleaf sign’. Additionally, endometriosis plaques are seen in bilateral ovaries and torus uterinum as hyperintense signal on axial fat-saturated T1 W images (**c**, **d**)

DIE lesions may infiltrate the anterior or posterior surface of uterus and extend inward to myometrium; as a result of this, anteversion or retroversion may occur in uterus. On the other hand, these lesions may cause infertility. Therefore, the extent of myometrial invasion and its proximity to endometrium are critical factors that dictate the treatment approach and should be reported. Besides, increased thickness due to the involvement of DIE lesions in torus uterinum, with T1 W-T2 W hypointense fibrous bands extending from torus uterinum to surrounding tissues (Fig. 7), and T1 W hyperintense endometrial implants may be observed (Fig. 8). In fact, these fibrous bands pull both ovaries and rectum towards torus uterinum, creating a condition described as the “cloverleaf” sign (Fig. 9). This has been shown to be associated with more complex surgery and more blood loss. [19, 27, 35].

In vaginal endometriosis, posterior vaginal cuff and/or fornix are the most common location of DIE involvement and frequently seen as fibrotic endometrial implants or polypoid lesions. A precise diagnosis of vaginal endometriosis with including depth of invasion is crucial for effective surgical planning [19].

**Fig. 10:**
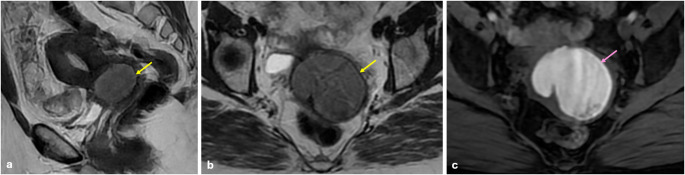
Cervical endometriosis. On sagittal (**a**) and axial T2 W (**b**) images show a hypointense (yellow arrows) and axial fat-saturated T1 WI (**c**) shows a hyperintense (pink arrow) 5 cm cystic endometriotic lesion in the cervix. The patient underwent a cone biopsy eight months ago

Cervical endometriosis is a rare condition for DIE that usually occurs after cervical trauma, such as biopsy, with implantation of endometrial fragments. DIE lesions may appear nodular, cystic, or polyploid. On MRI, these lesions appear hyperintense on T1 WI due to hemorrhage, also with hypointense cervical stroma on T2 WI [36] (Fig. 10).

### Posterolateral compartments

**Fig. 11:**
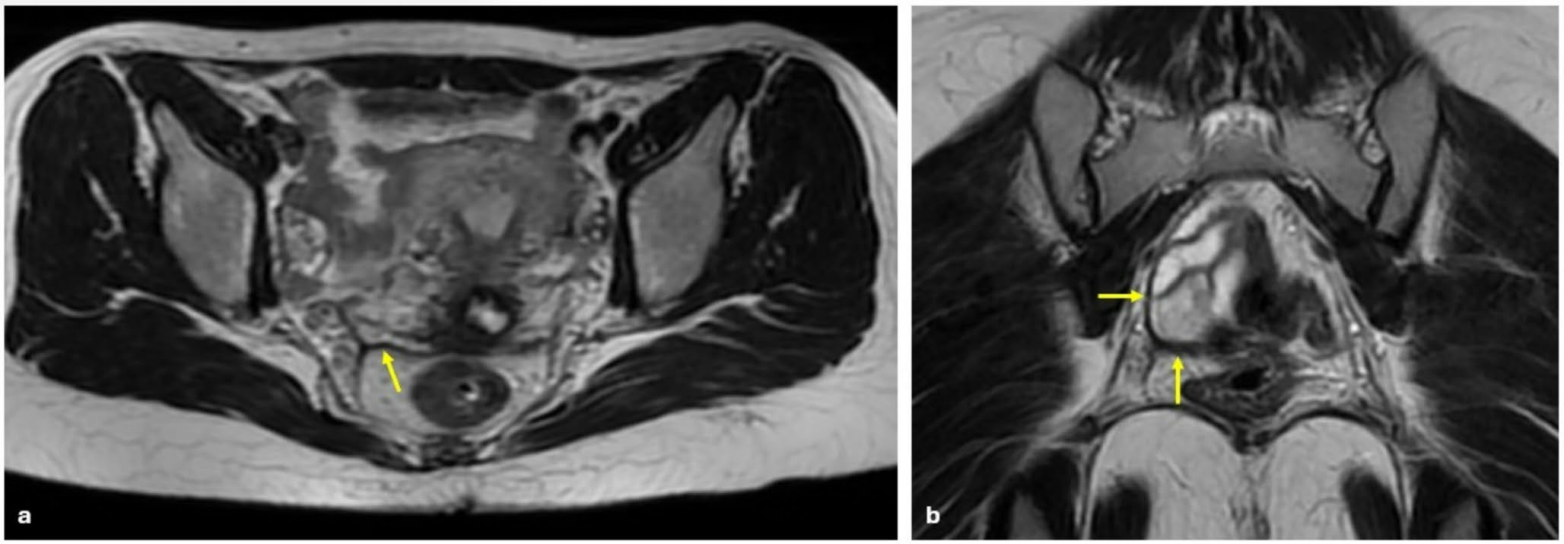
Deep infiltrating endometriosis with uterosacral ligament involvement. Right uterosacral ligament thickening (yellow arrows) is shown on axial (**a**) ve coronal (**b**) T2 W images

The posterolateral compartment includes the distal USL (> 2 cm from cervical insertion) (Fig. 11), sacro-recto-genital septum (SRGS), and pelvic wall (sacral roots, sciatic nerve, and internal iliac vessels) [30]. SRGS is the posterior retroperitoneal space extending beyond the mesorectum and is differentiated from the parametrium. USL forms the medial roof of SRGS. Hypogastric plexus is located within SRGS beneath the ureter and deep uterine vein [30, 37]. DIE lesions of SRGS have great importance due to the risk of rectal and bladder dysfunction, especially when the inferior hypogastric plexus is affected [37].

### Posterocentral compartment

**Fig. 12:**
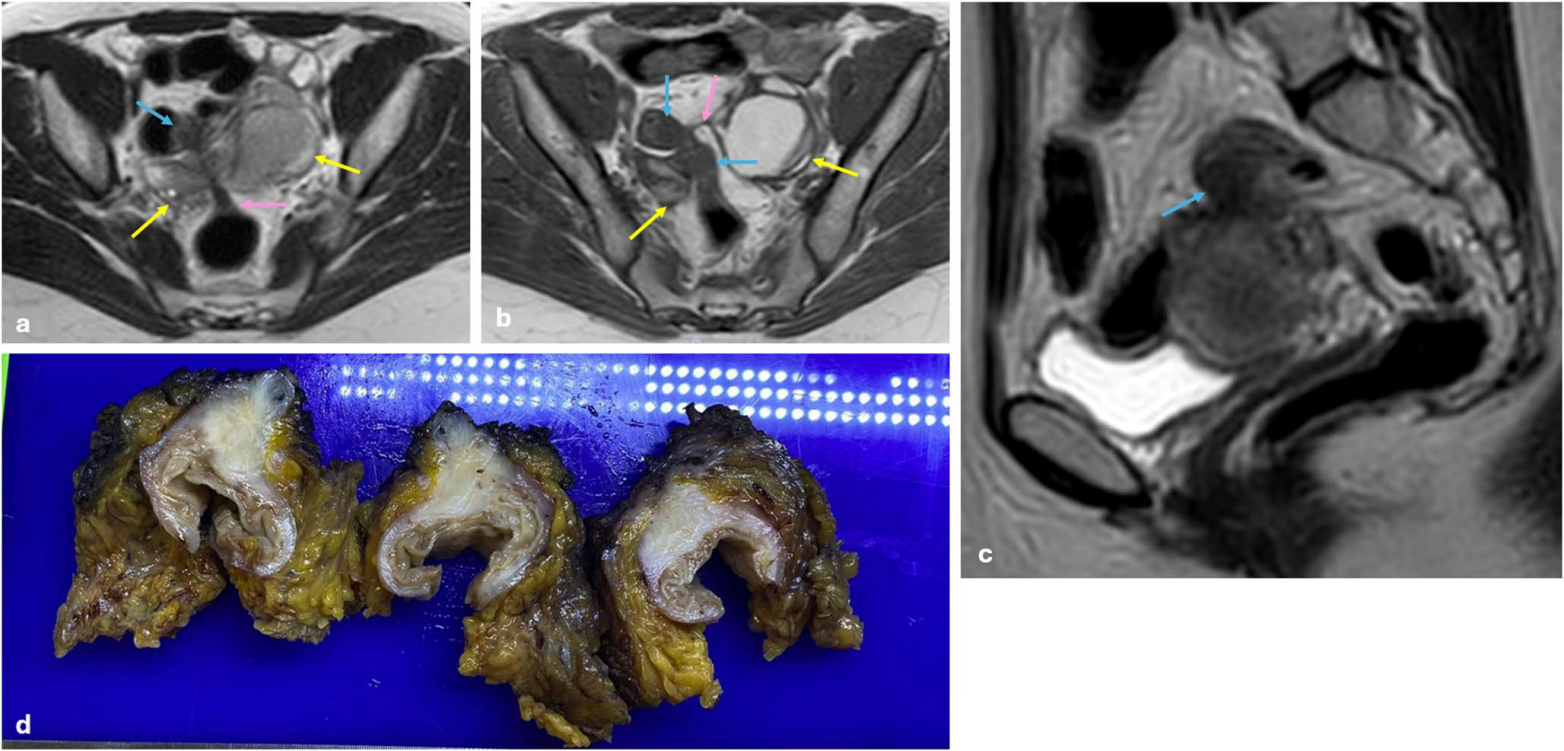
Deep infiltrating endometriosis with mushroom cap sign. In the right ovary, a 2 cm endometrioma and in the left ovary, a 4 cm endometrioma (yellow arrows) with fibrous bands extending from both endometriomas to the rectosigmoid colon (pink arrows) are shown on the axial T2 WI (**a**) and T1 WI (**b**). Significant thickening (blue arrows) is observed in the colon wall and clearly seen on sagittal T2 WI as a hypointense area-like mushroom cap (**c**). The wall thickening described in MRI is also seen in pathological specimens (**d**)

The posterocentral compartment includes rectum and rectosigmoid junction [30]. Rectosigmoid endometriosis is one of the crucial involvements of DIE. It is caused by superficial endometriosis lesions extending from peri-anatomical structures into the muscular layer of bowel wall. This situation is seen as hypointense nodules on T2 WI. Moreover, muscularis propria invasion may cause muscular hypertrophy and serosa puckering, which is known as the “mushroom cap” sign (Fig. 12). As a result, the lesion’s location- particularly its relationship to the peritoneal reflection and the distance from the anal verge-, the size and length of the involved bowel segments, and the depth of bowel wall invasion are vital for reporting, as they may alter surgical planning [19, 25, 35].

### Extrapelvic endometriosis

**Fig. 13:**
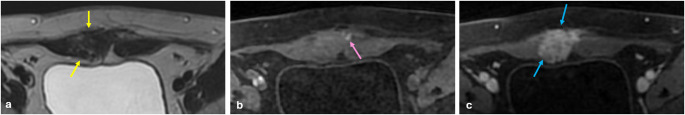
Abdominal wall endometriosis. On axial T2 WI (**a**), a heterogeneous hypo-isointense lesion within the right rectus muscle (yellow arrows), and on axial fat-saturated T1 WI (**b**), hyperintense hemorrhagic foci in the lesion (red arrow) are shown. On axial contrast-enhanced fat-saturated T1 WI (**c**), it is also shown that this lesion has enhancement (blue arrows). In a patient with a history of cesarean section, the diagnosis is abdominal wall endometriosis

**Fig. 14:**
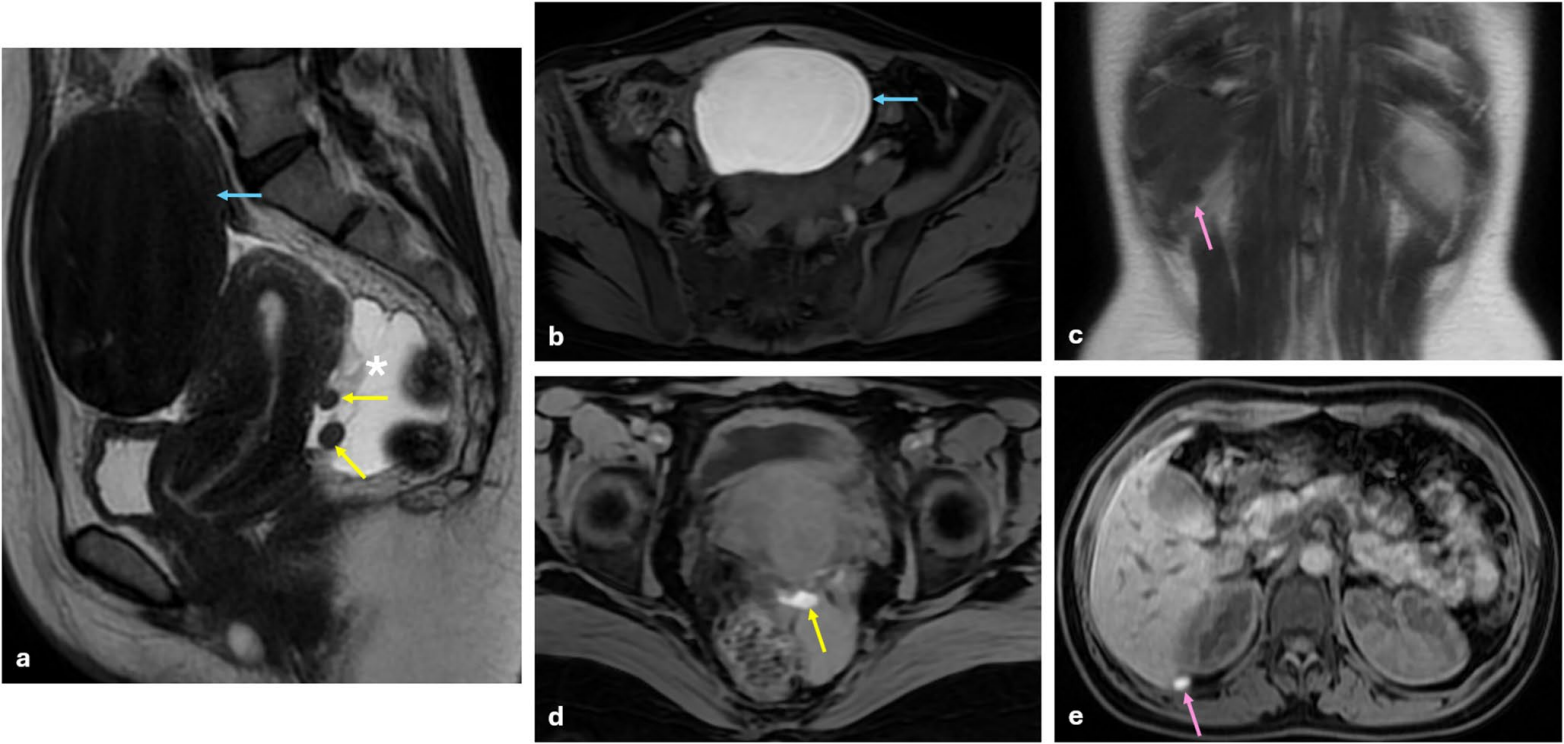
Extrapelvic endometriosis with ovarian endometrioma and deep pelvic endometriosis. In right ovary a 15 cm endometrioma (blue arrows) is shown on sagittal T2 WI and axial fat-saturated T1 WI (**b**). Additionally, two cystic endometriosis lesions measuring 1 cm and 1.5 cm (yellow arrows) are seen in the rectouterine space in sagittal T2 WI (**a**) and fat-saturated T1 WI (**d**), with fluid present in the Douglas pouch (asterisk). Furthermore, a similar endometriosis lesion measuring approximately 1 cm (pink arrows) is identified in the Morrison pouch in the coronal T2 WI (**c**) and axial fat-saturated contrast-enhanced T1 WI (**e**)

Extrapelvic endometriosis involvement sites and organs outside the pelvic endometriosis are defined separately and should be specified in the report. Extrapelvic endometriosis is similarly referenced in the SAR and ENDOVALIRM lexicon [19, 30]. Basically, extrapelvic region includes abdominal wall (Fig. 13), bilateral inguinal sites, sigmoid colon, cecum, ileum, appendix, ureters at the level of common iliac artery, upper abdomen site, thoracic site, and rarely other body parts. On MRI, extrapelvic endometriosis appears as similar imaging features to pelvic endometriosis lesions; hemorrhagic cysts or foci show a hypointensity on T2 WI and hyperintensity on T1 WI (Fig. 14), and fibrous bands or adhesions show hypointensity on both T1 and T2 W images [19, 25, 27].

## Conclusion

Endometriosis is a prevalent chronic inflammatory disease that particularly affects reproductive-aged women, significantly diminishing their quality of life and fertility. MRI can contribute to early and accurate diagnosis of superficial endometriosis, endometriomas, and DIE without requiring invasive procedures and radiation exposure. With the advantage of whole-body imaging, it is helpful in identifying millimetric endometriosis foci, especially in T1 W-FS series. Additionally, MRI plays a crucial role in assessing disease severity and provides great benefit in selecting optimum treatment and preoperative planning. Therefore, identifying endometriotic lesions is vital both in determining treatment and planning surgery.

## Data Availability

No datasets were generated or analysed during the current study.
